# Mobile App–Based Remote Patient Monitoring in Acute Medical Conditions: Prospective Feasibility Study Exploring Digital Health Solutions on Clinical Workload During the COVID Crisis

**DOI:** 10.2196/23190

**Published:** 2021-01-15

**Authors:** Sachin Shailendra Shah, Andrew Gvozdanovic, Matthew Knight, Julien Gagnon

**Affiliations:** 1 Huma Therapeutics London United Kingdom; 2 West Hertfordshire NHS Trust Watford United Kingdom

**Keywords:** mHealth, remote patient monitoring, digital health, COVID-19, service improvement, cost-effectiveness, monitoring

## Abstract

**Background:**

Digital remote patient monitoring can add value to virtual wards; this has become more apparent in the context of the COVID-19 pandemic. Health care providers are overwhelmed, resulting in clinical teams spread more thinly. We aimed to assess the impact of introducing an app-based remote patient monitoring system (Huma Therapeutics) on a clinician’s workload in the context of a COVID-19–specific virtual ward.

**Objective:**

This prospective feasibility study aimed to evaluate the health economic effects (in terms of clinical workload) of a mobile app on a telephone-based virtual ward used in the monitoring of patients with COVID-19 who are clinically ready for discharge from the hospital.

**Methods:**

A prospective feasibility study was carried out over 1 month where clinician workload was monitored, and full-time equivalents savings were determined. An NHS hospital repurposed a telephone-based respiratory virtual ward for COVID-19. Patients with COVID-19 in the amber zone (according to the National Health Service definition) were monitored for 14 days postdischarge to help identify deteriorating patients earlier. A smartphone-based app was introduced to monitor data points submitted by the patients via communication over telephone calls. We then comparatively evaluated the clinical workload between patients monitored by telephone only (cohort 1) with those monitored via mobile app and telephone (cohort 2).

**Results:**

In all, 56 patients were enrolled in the app-based virtual ward (cohort 2). Digital remote patient monitoring resulted in a reduction in the number of phone calls from a mean total of 9 calls to 4 calls over the monitoring period. There was no change in the mean duration of phone calls (8.5 minutes) and no reports of readmission or mortality. These results equate to a mean saving of 47.60 working hours. Moreover, it translates to 3.30 fewer full-time equivalents (raw phone call data), resulting in 1.1 fewer full-time equivalents required to monitor 100 patients when adjusted for time spent reviewing app data. Individual clinicians spent an average of 10.9 minutes per day reviewing data.

**Conclusions:**

Smartphone-based remote patient monitoring technologies may offer tangible reductions in clinician workload at a time when service is severely strained. In this small-scale pilot study, we demonstrated the economic and operational impact that digital remote patient monitoring technology can have in improving working efficiency and reducing operational costs. Although this particular RPM solution was deployed for the COVID-19 pandemic, it may set a precedent for wider utilization of digital, remote patient monitoring solutions in other clinical scenarios where increased care delivery efficiency is sought.

## Introduction

### Background

Over the last decade, the world has seen a surge in the creation of virtual wards, wherein patients are managed remotely [1]. With an aging population [2], the demand for health care has started to exceed supply, thereby causing significant strain on service provision [3]. The need for a solution that reduces the burden on both primary and secondary health care services is therefore paramount. 

### Economic Benefits of Remote Patient Monitoring

In the UK, as part of the National Health Service’s (NHS) Long Term Plan, digital initiatives are being reviewed for their potential integration into the current national health care system, and primary care services are at the forefront of this movement [4]. It is thought that web-based, digital general practice consultations and redesigned hospital support will reduce outpatient appointments by up to a third [4]. This could reduce patient trips to the hospital by approximately 30 million each year and, in turn, save the NHS over £1 billion (~US $1.35 billion) annually in new expenditure averted [4]. Although the use of remote patient monitoring (RPM) has typically revolved around the management of chronic diseases (eg, diabetes) and perioperative care (eg, orthopedic surgery), the expansion of RPM to wider patient groups promises additional benefits to both health care professionals and patients. Evidence regarding clinical outcomes associated with RPM is limited; however, there is overwhelming evidence of its economic benefits; for example, a reduction in clinician workload allowing the redirection of services to more demanding environments. 

In the South Eastern Trust of Northern Ireland, the use of virtual wards was found to create cost-avoided savings of £ 8,804,529 (~US $11,940,922) over a 3-year period. This case study evaluated a virtual ward operational in 3 locations encompassing patients with chronic health conditions such as respiratory disorders, heart failure, and diabetes [5]. Over the course of the 3-year review period, an estimated 812 hospital admissions were avoided, and 447 episodes of care were provided by the virtual ward service, thereby effecting a total saving of approximately 4547 bed days. The operational costs over the 3 years are approximated at £ 566,273 (~US $768,106), which equates to a total net saving of £ 8,238,256 (~US $11,175,433) [5].

Similar evidence was also reported by the Healthcare at Home team who, in 2014, effected a reduction in the number of bed days by 130,000 [1] via the implementation of virtual wards. As such, regardless of whether there are any clinical benefits, it is clear that RPM has significant economic benefits, with multiple studies showing cost-saving outcomes following the implementation of a virtual ward [1,5]. 

### RPM in Disease States

Considerable evidence supports the use of RPM, although with varying levels of robustness, in the care of chronic diseases such as heart failure, chronic obstructive pulmonary disorder (COPD), and frailty [6,7]. However, to date, there has been little research on the management of acute illnesses or, specifically, respiratory illnesses in this setting. Differences between the management of chronic and acute illnesses can be striking. Therefore, when attempting to extrapolate the economic impact of virtual wards on chronic disease management to acute disease management, we may expect to encounter some disparity.

In a previous study evaluating the use of an RPM solution in the care of exacerbation of COPD and chronic heart failure, Isaranuwatchai et al [8] studied the economic impact of remote monitoring symptoms to detect early deterioration of patient health. It is, therefore, somewhat suited to an evaluation in the context of monitoring of acute conditions. This study showed a potential reduction of 68% and 35% in the number of emergency room (ER) visits and hospitalizations, respectively, between the 3-month pre– and post–RPM intervention periods. The average ER visit cost was reduced from CA $243 (~US $191) at the baseline to CA $67 (~US $53) during the 3-month follow-up and from CA $3842 (~US $3023) to CA $1399 (~US $1100) for hospitalization [8]. This result demonstrates that an RPM solution can not only free-up resources but also lead to a less resource-heavy and, therefore, less-expensive visit or admission in the context of acute care.

### RPM During the COVID-19 Pandemic

Health systems worldwide are currently facing an unprecedented challenge that is rapidly transforming the ways in which clinical care is provided. As of July 2020, the total number of confirmed cases of COVID-19 worldwide was approximately 15,201,000, with an estimated total of 623,000 related deaths [9]. At the time of this research project, no current vaccine nor effective treatment was available, and minimizing the risk of exposure was the mainstay of intervention at a population level [10].

Thus, we find ourselves using health care systems that are fundamentally rooted in face-to-face interactions and managing a disease by implementing preventive measures such as social distancing and hygiene education—a challenging combination of contrasts. Many institutions are, therefore, turning to connected care or digital health solutions such as virtual wards, RPM, and telemedicine [11].

Traditionally, the speed at which these digital interventions have been introduced has been slow [12]. The COVID-19 pandemic, however, has led to a transformation in the mobile health (mHealth) landscape, with institutions choosing to quickly implement mHealth solutions and adapt rapidly [13]. A report by Mann et al [13] describes the feasibility and impact of a video-enabled telemedicine solution at the epicenter of the COVID-19 outbreak in New York. The study, carried out in conjunction with NYU Langone Health, demonstrated the impact of this mHealth solution across 25 locations. Among the various outcomes evaluated, they reported an increase in the number of daily telemedicine visits from 369.1 to 866.8 in urgent care settings and from 94.7 to 4209.3 in nonurgent care settings, of which 56.2% and 17.6% of the visits, respectively, were related to COVID-19. Overall, clinicians found the existing telemedicine solution to be a useful tool in diverting patients from the ER, in order to prioritize those who needed acute care and thus minimize the risk of COVID-19 exposure [13].

Owing to the unique impact of COVID-19 on health care systems, there is limited evidence to reflect on the economic effects of RPM on pandemics of this kind. Consequently, COVID-19 provides an opportunity to explore the economic impact of widespread implementation of RPM for acute care. These analyses may also be applicable to other pandemics and standard practices alike, as most research investigates the use of RPM for exacerbations of chronic disease states and their associated illnesses. These analyses are generally handled with well-established processes with known protocols in place. Such economic evaluations would, therefore, not take into account the potential impact of virtual wards on the following: reduction of viral transmission (and the subsequent knock-on effect of reduced sickness on the economy), increase in the efficiency of resource use (such as from those high-risk individuals who would not be available for front-line work), or reduction in the utilization of other resource such as personal protective equipment [14,15]. 

With the increasing popularity of digital technologies providing mobile app–based solutions for digital health, virtual wards have somewhat undergone an overhaul and are now able to provide clinical teams with real-time data to better manage their patients. Clinicians now have greater visibility over their patients and improved communication pathways with other health care professionals, all of which have been shown to improve the efficacy of virtual wards within mHealth solutions and, thus, enhance health care in general [16].

### Study Aims

The aim of this study was to evaluate the operational and consequent economic benefits of app-based RPM as a supplement to the existing telephone-based virtual wards in the context of the COVID-19 pandemic.

## Methods

An NHS district hospital, in partnership with NHS-X and Huma Therapeutics, utilized its existing respiratory virtual ward as a temporary COVID-19 virtual ward. This virtual ward was initially designed to support inpatients who were experiencing exacerbated common respiratory conditions and met the criteria for early discharge. In the wake of COVID-19, this ward was used to monitor medium-risk patients (as per the NHS COVID-19 guidelines) who were ready to be discharged from the hospital. These patients needed to be monitored for 14 days to ensure no deterioration occurred but were deemed clinically safe to be discharged. Patients had the option of being monitored solely via telephone calls (cohort 1) or via a combination of mobile app and telephone calls (cohort 2).

Patients in cohort 1 followed a structured telephone call plan and would be followed-up at regular intervals via phone calls. Typically, a member of the respiratory team (ie, consultant, physiotherapist, or physiologist) would call patients in cohort 1 on days 1, 2, 3, 4, 5, 7, 9, 11, and 14. Patients were discharged after 14 days, if deemed clinically safe to do so. Phone calls were made to assess the patients’ symptoms and functionality in relation to daily activities and, if possible, record any vital signs the patient may have taken.

**Figure 1:**
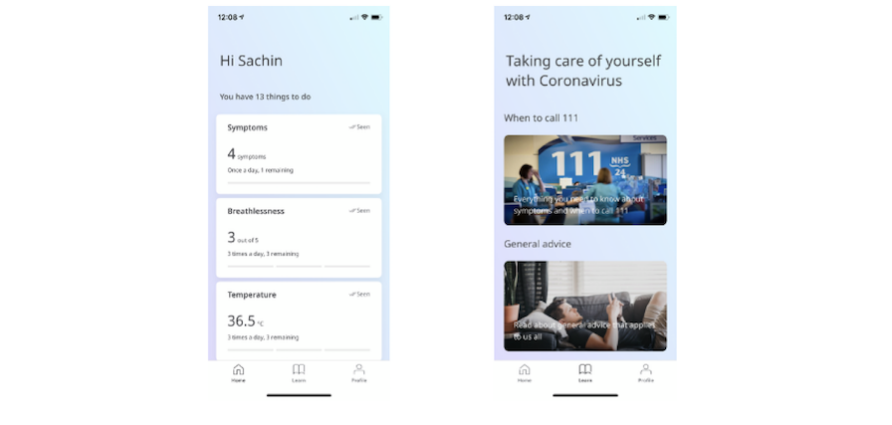
Screenshots of the mobile app (Huma Therapeutics) used by the study patients.

Patients in cohort 2 were virtually onboarded to the Huma Therapeutics app. They were instructed via the app (Figure 1) to submit the following data on a daily basis: heart rate (obtained via photoplethysmography technology embedded in the app); oxygen saturation (obtained via a pulse oximeter wirelessly connected to the app or by manual entry); body temperature (obtained via a digital thermometer connected to the app or by manual entry); any symptoms (Textbox 1) experienced; and breathlessness measured using a single-question questionnaire (“How breathless are you when walking around or walking upstairs?”) created by the clinicians involved in the project, which was scored on a scale of 1-5, with 1 being the least and 5 being the worst). This data was manually transcribed to a variety of electronic health records by populating a premade template. Based on the data submitted, a member of the clinical team would decide on whether a phone call was required or not. As per cohort 1, patients were monitored for 14 days, after which, if clinically safe, they were discharged.

The aim of this study was to investigate the operational and economic impact of an app-based RPM tool on clinician workload in a telephone-based virtual ward, in the context of managing patients with COVID-19. This prospective feasibility study was carried out over a 1-month period, during which clinician workload (ie, number and length of phone calls) was monitored and full-time equivalent (FTE) savings were equated. Clinical outcomes were defined as mortality and readmissions, simply as a basis to confirm noninferiority. Moreover, some informal qualitative information from clinician end-users was collected based on phone call quality and end-user feedback via unstructured telephone interviews.

**A list of symptoms available on the app for patients to choose from and submit to their care team.**

**List of symptoms:**

- Fever
- Cough
- Shortness of breath
- Nausea
- Loss of taste
- Loss of smell
- Vomiting
- Chest pain/tightness
- Headache
- Heart palpitations
- Dizziness
- Loss of consciousness

## Results

Over a 1-month period, a total of 56 patients were enrolled into cohort 2 (app + telephone). These patients had a clinical or laboratory diagnosis of COVID-19. Of the 56 patients, 31 (55%) were male and 25 (45%) were female. The mean age of the study participants was 64 years (age range 21-79 years). Of these 56 patients, 40 (71%) patients used an iPhone, and the remainder 16 (29%) used an Android device. Clinical staff were hired for 12 hours a day and the service was available 7 days a week. We do not have the data on the number of patients enrolled into cohort 1 at this time.

Over the course of the monitoring period, we found that patients in cohort 2 (app + telephone) received significantly fewer phone calls (mean 4, SD 0.701) than those in cohort 1 (telephone only; mean 9, SD 1.13). In addition, the mean phone call time for patients in cohort 1 was 8.5 minutes, which was similar to the mean phone call time in cohort 2 (data not available).

**Figure 2:**
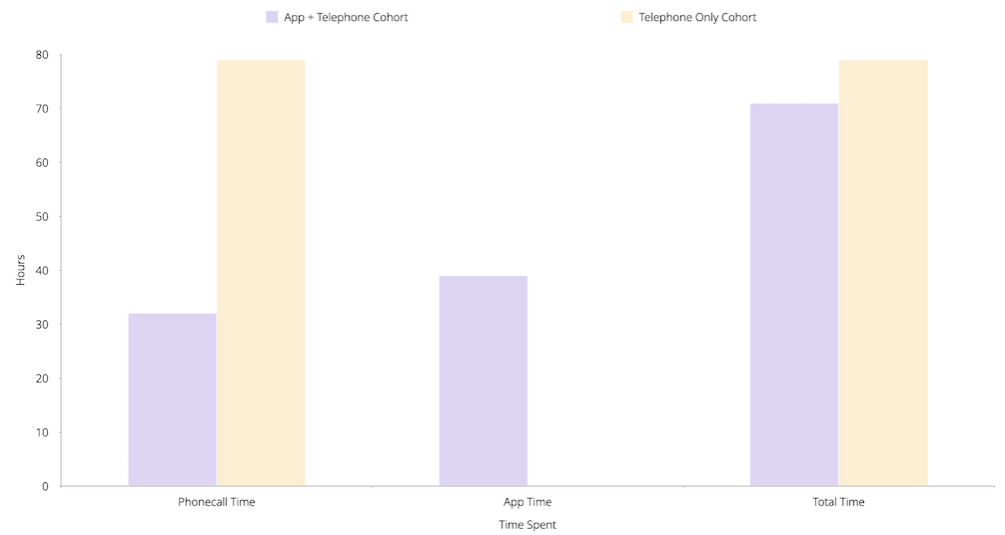
Difference in time spent remotely monitoring 56 patients between the 2 cohorts.

The total time spent on phone calls for all 56 patients in cohort 2 was 31.73 hours. Based on the mean phone call time, the total time spent on phone calls for 56 patients in the cohort 1 model would be 79.33 hours. This equates to a 60% (47.60 hours) reduction from cohort 1 to cohort 2 (Figure 2). During this period, 7 clinicians monitored the 56 cohort patients. In terms of health economics, we observed a reduction of 3.30 FTE (ie, the number of clinicians reviewing these 56 patients). Each clinician spent an average of 10.9 minutes a day reviewing data of patients in cohort 2, resulting in a total time of 38.68 hours spent on the clinician dashboard. The FTE adjusted for time spent reviewing data was 1.1 per 100 patients; that is, for every 100 patients monitored in cohort 2, 1 less clinical personnel was needed compared to cohort 1.

Clinically, among the 56 patients reviewed, there were no mortalities and readmissions 14 days after discharge, indicating noninferiority of the model. It is worth noting that, due to the small patient sample size, the lack of observed mortalities and readmissions may not indicate a genuine impact of remote monitoring on these particular outcome metrics.

Informal qualitative feedback from clinicians held the solution in high regard. Qualitative data indicated that the app was emotionally well received by patients (“It’s like an extension of the human touch.”) and that the particular solution adopted was easy to use (“The Medopad patients have been much quicker and easier to deal with than non-Medopad virtual ward patients.”)

## Discussion

### Summary

This prospective feasibility study highlights the impact a digital health solution can add to existing care services. In areas where large volumes of patients need to be monitored, an app-based tool can considerably reduce the time needed by the clinical team to manage the said cohort. Our small-scale, pilot study shows that the introduction of an app to supplement existing care pathways reduces the amount of time a clinician ends up spending to manage patients. Extrapolated economically, for every 100 patients enrolled into the virtual ward, you would need one less member of the clinical team to manage the group compared with the traditional phone call–only management style.

The COVID-19 pandemic has brought to light significant challenges concerning the infrastructure of health care provision, especially in acute, high-demand settings. Technology has advanced exponentially over the last few decades; however, digital solutions for monitoring health are still in their infancy. Digital, app-based solutions have historically shown large economic benefits with the additional potential for large-scale clinical benefit. They allow for greater reallocation of resources in terms of clinical personnel work distribution, allowing health professionals to better manage their already overburdened schedules. 

### Strengths and Limitations

Although this study was based on a small sample, the results clearly show the need for more robust and greater powered studies. This study depicts an improvement in service delivery; therefore, larger, more generalizable studies could not only compound these findings but also help demonstrate this positive effect in different care environments. If the study findings are generalizable, it may be concluded that an mHealth pathway could reduce the number of doctors needed to monitor these patients, if faced with a second wave or a similar pandemic. These studies need to accurately compare the time spent utilizing digital solutions with that spent monitoring patients by using in-person pathways, to ascertain a true economic representation of the change in the pathway. In addition, more formal, qualitative feedback needs to be obtained, not only from clinicians but also from patients, to review the psychological impacts of such digital health solutions. Further parallel studies should be run to accurately gauge clinical outcomes of mHealth solutions compared to traditional methods of patient monitoring.

### Comparison With the Existing Literature

The existing literature clearly demonstrates the vast benefits across clinical and operational outcomes. However, majority of these studies are pilot studies and there are yet to be large landmark studies confirming the efficacy of digital solutions in the management of patients. In regard to the UK, the NHS clearly explains the need for an increase in digital services; however, evidence of this occurring, or even working, is nonexistent. COVID-19 has prompted fast deployment of mHealth solutions; over time, this evidence will start to come to fruition. This study highlights the value of digital tools in novel disease states with clear potential advantages if applied to different care pathways.

### Implications for Research or Practice

The findings of this study highlight the benefits of mHealth solutions and allow health care providers to introduce similar solutions to help manage future waves of COVID-19 and other pandemics. Clinical teams should heed the benefits displayed and implement these time- and cost-saving services into the everyday infrastructure. This would not only mean services are better prepared for future waves or pandemics but can also improve the operation of their day-to-day patient loads. As demand for health care continues to rise, any tool that can help reduce clinical team workload and allow for more patients to be seen with the same number of staff would be regarded as an invaluable tool.

## Abbreviations

COPD: chronic obstructive pulmonary disorder
ER: emergency room
FTE: full-time equivalent
mHealth: mobile health
NHS: National Health Service
RPM: remote patient monitoring
